# Bullying victimization in children and adolescents and its impact on academic outcomes

**DOI:** 10.1192/j.eurpsy.2022.388

**Published:** 2022-09-01

**Authors:** R. Vadukapuram, C. Trivedi, Z. Mansuri, K. Shah, A. Reddy, S. Jain

**Affiliations:** 1 Icahn School of Medicine at Mount Sinai, Psychiatry, New York, United States of America; 2 Texas Tech University Health Science Center at Permian Basin, Psychiatry, Midland, United States of America; 3 Boston Childrens Hospital, Psychiatry, MA, United States of America; 4 Griffin Memorial Hospital, Psychiatry, Norman, United States of America; 5 Virginia Tech Carilion School of Medicine, Psychiatry, Roanoke, United States of America

**Keywords:** bullying, mental health, Child Psychiatry

## Abstract

**Introduction:**

Bullying is a serious problem in schools because of the negative impact on a child’s educational outcomes, especially academic achievement. However, the underlying mechanisms and causes are unknown.

**Objectives:**

To evaluate the educational outcomes, and psychiatric comorbidities in children and adolescents who are victims of bullying

**Methods:**

We used 2018–2019 Nationwide Survey of Children’s Health (NSCH) dataset for the study. The participants were children and adolescents (age: 6-17 years, n = 42,790). Data was stratified into two groups: 1) never bullied 2) bullied more than once. Prevalence of different educational outcomes were compared between the groups.

**Results:**

In the never bullied group 21,015 participants were included, and in the bullied more than once group 21,775 participants were included. More females were in the bullied group compared to never bullied group (50.4% vs 47.5%, p=0.006). More White non- Hispanic individuals were in bullied group in contrast to never bullied group (56.7% vs 43.9%, p< 0.001). Individuals whose health status was fair, or poor were bullied more (2.4% vs 1.4%, p=<0.001). Individuals in bullied group were more likely to be repeating the grades compared to the never bullied group (7.1% vs 5.9%, p:0.039). Individuals who were missing >=11 school days, and sometimes or never engaged in school were observed more in bullied group compared to never bullied group (5.9% vs 3.2% and 20.3% vs 10.6% p < 0.001).

**Conclusions:**

Our findings suggest that bullying victimization could be a risk factor and associated with decreased academic outcomes.

**Disclosure:**

No significant relationships.

